# GERONTOLOGICAL PEDAGOGY: PASSION, PARADOX, AND PROMISE

**DOI:** 10.1093/geroni/igz038.234

**Published:** 2019-11-08

**Authors:** Sarah J Hahn, Rona J Karasik

**Affiliations:** 1 Mercy College, Dobbs Ferry, New York, United States; 2 St. Cloud State University, Saint Cloud, Minnesota, United States

## Abstract

Gerontology programs in the United States are on the decline despite the need for trained professionals who can serve a growing aging population (Pelham, Schafer, Abbott, & Estes, 2012). For many students, a gerontology course may be their only formal exposure to the concepts of aging. The development of gerontological pedagogy is important, but there is limited knowledge about what is being taught around the nation and to what extent course content reflects the current scope of the discipline. This symposium explores the role of individual instructors, the larger environment, and the efforts of organizational level criteria (i.e., AGHE standards to advance the field) to promote higher-quality gerontological education. The first presenter reports dissertation findings that examined college students’ self-perceptions of aging and how stereotypes impact them even after taking an introduction to gerontology course. The second presenter discusses the need for service-learning and community engagement in gerontology, while reporting on theoretical and practical suggestions, as well as potential pitfalls to avoid. The third presenter reports on student evaluations in a Master of Science program before and after the implementation of the Association for Gerontology’s proposed competencies. And our final presenter provides insights on applied perspectives and pedagogical approaches in and out of the classroom, including pitfalls and possibilities. Our discussant brings our ideas together to report on the discipline of gerontology and our potential to advance to the next level of pedagogical strategies.

